# Perfectionism and practice procrastination in piano majors: a moderated mediation model of music performance anxiety and self-efficacy

**DOI:** 10.3389/fpsyg.2026.1786554

**Published:** 2026-05-14

**Authors:** Xuemei Zhang

**Affiliations:** Department of Music, Jinzhong University, Jinzhong, Shanxi, China

**Keywords:** avoidance behavior, music performance anxiety, music students, perfectionism, practice procrastination, self-efficacy

## Abstract

**Introduction:**

Practice procrastination is a commonly observed yet understudied issue in music education, including among piano majors who face sustained demands for daily deliberate practice. Perfectionism, a common personality trait in music students, may be associated with practice procrastination, and MPA may serve as an intermediary in this relationship by translating perfectionistic concerns into avoidance-oriented practice behavior. However, this mechanism remains empirically untested, and how individual differences moderate this process is unclear.

**Methods:**

This cross-sectional study examined these relationships using a moderated mediation model among 156 Chinese undergraduate piano majors (67.9% female; *M* age = 20.35 years, *SD* = 1.42). Participants completed the Frost Multidimensional Perfectionism Scale, Kenny Music Performance Anxiety Inventory-Revised (K-MPAI-R), General Self-Efficacy Scale, and an adapted Practice Procrastination Scale. Data were analyzed using SPSS 26.0 and PROCESS macro (Model 14) with 5,000 bootstrap samples.

**Results:**

Perfectionism was positively associated with practice procrastination both directly and indirectly, with MPA serving as an intermediary variable in this relationship. Self-efficacy moderated the association between MPA and procrastination, such that higher self-efficacy attenuated or eliminated the positive link between anxiety and procrastination. The moderated mediation analysis supported the conditional nature of the indirect pathway, with its strength varying as a function of self-efficacy level.

**Discussion:**

These findings suggest a cognitive-emotional-behavioral pathway in which perfectionism is associated with practice procrastination, with MPA appearing to function as an emotional intermediary and self-efficacy serving as a potential protective factor. The results offer empirically informed guidance for music educators seeking to identify at-risk students and develop targeted interventions, and they suggest that enhancing self-efficacy may help disrupt the anxiety–procrastination association.

## 1 Introduction

Piano learning requires long-term commitment and systematic practice (McPherson, 2022). However, many piano students suffer from procrastination, repeatedly postponing or avoiding practice despite knowing it is necessary. This procrastination not only hinders professional skill development but can also exacerbate pre-performance anxiety, creating a vicious cycle. Passarotto et al. (2023) pointed out that dysfunctional practice behavior is a significant risk factor for performance-related injuries in musicians; Cheng and Southcott (2023) also emphasized the importance of motivation and self-regulation in piano practice. The consequences of procrastination are multifaceted: in the short term, students may perform poorly in exams or performances due to insufficient preparation; in the long term, persistent procrastination hinders technical progress, weakens students’ confidence in music learning, and even affects their career trajectory. Therefore, understanding the causes of procrastination and finding effective intervention strategies is of significant practical importance.

Perfectionism is a commonly observed personality trait among music students (Stoeber and Eismann, 2007), and a meta-analysis by Curran and Hill (2019) documented increasing levels of perfectionism among younger cohorts. Previous studies have found a close association between perfectionism and musical performance anxiety (MPA): Mor et al. (1995) confirmed that perfectionism is an important predictor of performance anxiety among professional performing artists; Kenny et al. (2004) found a correlation between perfectionism and MPA and occupational stress among opera artists; Butković et al. (2022) confirmed a positive correlation between maladaptive perfectionism and MPA among Croatian musicians; and Yang et al. (2022) also verified this relationship among Chinese piano students.

Although the relationship between perfectionism and MPA has been well studied, how perfectionism influences practice procrastination remains a lack of empirical evidence. “Practice procrastination” as an independent construct has not received sufficient attention in music education. Furthermore, what are the psychological mechanisms by which perfectionism influences practice procrastination? What factors might moderate this process? These questions remain to be explored.

### 1.1 Theoretical basis and research hypotheses

#### 1.1.1 Perfectionism and practice procrastination

Perfectionism is a personality tendency characterized by setting excessively high standards and critical self-evaluation. Frost et al. (1990) proposed a multidimensional model of perfectionism, including six dimensions, which Stöber (1998) simplified to four core dimensions. Hewitt and Flett (1991) distinguished between self-oriented, other-oriented, and socially prescribed perfectionism from an interpersonal perspective. Among these, “concern about mistakes” and “doubt about actions” are considered core components of maladaptive perfectionism. This study focuses on maladaptive perfectionism, hereinafter referred to as “perfectionism.”

In the field of procrastination research, Flett et al. (1992) found a significant positive correlation between socially prescribed perfectionism and academic procrastination; Steel (2007) meta-analysis supports the view that perfectionism is an important predictor of procrastination; Sirois et al. (2017) meta-analysis further confirmed the differentiated associations between various dimensions of perfectionism and procrastination. Theoretically, perfectionists may employ procrastination as a self-protective strategy due to an excessive fear of failure (Yosopov et al., 2024). Therefore, we propose: Given the cross-sectional design of this study, the hypotheses are framed in terms of statistical associations rather than causal determination. The term “predicts” is used throughout to denote statistical prediction within a regression framework, not temporal or causal precedence.

*Hypothesis* 1 (H1): Perfectionism significantly and positively predicts practice procrastination.

#### 1.1.2 Mediating role of music performance anxiety

MPA is a persistent and context-dependent anxiety experience associated with musical performance that encompasses cognitive symptoms (e.g., worry about evaluation and fear of memory slips), physiological arousal (e.g., trembling, increased heart rate), and behavioral manifestations including avoidance of practice and performance situations (Kenny, 2011; Irie et al., 2023). Importantly, MPA is not limited to the moment of public performance; anticipatory anxiety can extend into practice and preparation phases (Irie et al., 2023). The K-MPAI-R used in this study captures a broad range of these components, including proximal somatic anxiety, worry and dread, and depression/hopelessness dimensions (Kenny, 2009), and the total score was used as a composite indicator reflecting the global severity of performance-related anxiety across different phases of musical engagement.

Perfectionism is a significant risk factor for MPA. Patston and Osborne (2016) found a persistent strong positive correlation between MPA and perfectionism in school-aged music students; Kobori et al. (2011) confirmed that perfectionist cognition is associated with coping styles and performance anxiety among Japanese musicians; Dobos et al. (2019) found that perfectionism dimension and social phobia jointly predict MPA; Gök and Yalçınkaya-Alkar (2025) verified the interaction between perfectionism, negative evaluation fear, and MPA in music conservatory students; Spahn et al. (2024) found differences in personality traits among musicians with different MPA types.

On the other hand, anxious individuals tend to use avoidance strategies to cope with threatening situations. Biasutti and Concina (2014) found that coping strategies significantly predicted MPA levels; Passarotto et al. (2023) confirmed a significant association between anxiety and practice behavior in piano students; Sokoli et al. (2022) found that pre-competition anxiety and catastrophic thinking in classical music students predicted self-evaluated performance quality. From an emotion regulation perspective, anxious individuals tend to achieve short-term emotional relief through avoidance behavior, and procrastination is an avoidance strategy (Sirois and Pychyl, 2013; Sirois, 2023). Notably, Sirois (2023) further proposed a stress context vulnerability model in which stressful contexts increase the risk of procrastination because it functions as a low-resource means of avoiding aversive task-related emotions, a pattern that may be particularly relevant to the high-pressure evaluative context of piano practice. Therefore, maladaptive perfectionism (a cognitive characteristic) may lead to practice procrastination (avoidance behavior) by triggering MPA (emotional response).

*Hypothesis* 2 (H2): MPA may serve as an intermediary variable in the association between perfectionism and practice procrastination.

#### 1.1.3 The moderating role of self-efficacy

Self-efficacy is an individual’s belief in their ability to complete a specific task (Bandura, 1977). Luszczynska et al. (2005) validated the cross-cultural validity of general self-efficacy scales. In the field of music, McPherson and McCormick (2006) found that musical self-efficacy is an important factor in predicting musical performance; Ritchie and Williamon (2011) developed a musical self-efficacy scale that distinguishes between learning and performance, and confirmed its predictive validity for performance quality (Ritchie and Williamon, 2012); González et al. (2018) confirmed that self-efficacy can buffer the negative impact of anxiety on performance quality.

In procrastination research, Seo (2008) and Huang et al. (2023) both found that self-efficacy plays an important role in the relationship between perfectionism and procrastination. In the field of music, Wang and Yang (2024) verified the moderating role of self-efficacy between MPA and career aspirations among Chinese music students; Arbinaga Ibarzábal (2023) found that resilience behavior in music students was associated with perfectionism and self-efficacy. Varela et al. (2016) emphasized the importance of self-regulation in music learning, a construct closely related to self-efficacy insofar as individuals with stronger efficacy beliefs tend to engage more effectively in self-regulatory processes such as goal-setting and self-monitoring (Bandura, 1977).

This study argues that self-efficacy primarily influences an individual’s behavioral coping choices when facing anxiety. Maladaptive perfectionists, due to their cognitive characteristics (excessive focus on errors, doubt about actions), are prone to anxiety responses in performance situations, and self-efficacy is unlikely to disrupt this cognitive-emotional connection; however, students with high self-efficacy, even when feeling anxious, still believe they can overcome difficulties and are more inclined toward problem-focused strategies rather than avoidance. This study positions self-efficacy as a moderating variable on the second segment of the indirect pathway (i.e., the link between MPA and practice procrastination) rather than the first segment (i.e., the link between perfectionism and MPA), proposing:

*Hypothesis* 3 (H3): Self-efficacy moderates the relationship between music performance anxiety and practice procrastination.

*Hypothesis* 4 (H4): Self-efficacy moderates the second segment of the indirect pathway from perfectionism to practice procrastination through MPA.

### 1.2 Study aim and research hypotheses

Drawing on the theoretical analysis above, the aim of this study was to examine how perfectionism is associated with practice procrastination and to clarify the psychological mechanisms and boundary conditions underlying this association among piano majors. A moderated mediation model was proposed in which perfectionism is directly associated with practice procrastination and indirectly linked to it through MPA, with self-efficacy moderating the second segment of the indirect pathway (i.e., the MPA–procrastination link). This model integrates cognitive, emotional, and behavioral perspectives. The four hypotheses derived from the preceding theoretical review are as follows: perfectionism is positively associated with practice procrastination (H1); MPA serves as an intermediary variable in the association between perfectionism and practice procrastination (H2); self-efficacy moderates the relationship between MPA and practice procrastination (H3); and self-efficacy moderates the indirect association of perfectionism with practice procrastination through MPA (H4).

## 2 Methods

### 2.1 Participants

Participants were recruited via an online questionnaire platform (Wenjuanxing) using convenience sampling from undergraduate piano students at Chinese universities. Inclusion criteria: (1) Undergraduate students majoring in piano performance or music education (piano concentration); (2) Students with a fixed weekly practice requirement. Exclusion criteria: (1) Students not majoring in piano; (2) Students with a history of mental illness diagnosis.

Fritz and MacKinnon (2007) provided sample size guidelines for simple mediation models, recommending 71 to 148 participants for achieving 0.80 power with the bias-corrected bootstrap test under medium-to-large effect size combinations. Although these benchmarks do not directly apply to moderated mediation designs involving interaction terms, the present sample of 156 participants meets or exceeds these recommendations. A dedicated power analysis for conditional process models was not conducted, and this is acknowledged as a limitation. 200 questionnaires were distributed through music departments of multiple universities, and 156 valid questionnaires were collected, with an effectiveness rate of 78.0%. The sample included comprehensive universities and normal universities. Exclusion reasons included: 16 questionnaires with too short a response time (<3 min), 11 questionnaires with incorrect answers to attention test questions, and 17 questionnaires from non-piano majors. The sample characteristics are as follows: In terms of gender, there were 106 females (67.9%) and 50 males (32.1%); the age range was 18–24 years old, with an average age of 20.35 years (*SD* = 1.42); the grade distribution was as follows: 38 freshmen (24.4%), 42 sophomores (26.9%), 45 juniors (28.8%), and 31 seniors (19.9%); the average years of piano study was 12.68 years (*SD* = 3.25); in terms of major, 68 students (43.6%) majored in piano performance, and 88 students (56.4%) majored in music education (piano concentration).

### 2.2 Research tools

The Chinese versions of all instruments used in this study were obtained from previously published and validated translations. The Chinese version of the General Self-Efficacy Scale was translated and validated by Wang et al. (2001) with demonstrated reliability and unidimensional structure in Chinese samples. The Chinese version of the Frost Multidimensional Perfectionism Scale has been widely used in Chinese psychological research with established psychometric properties. The Chinese version of the K-MPAI-R was used following standard translation procedures and has been employed in prior research with Chinese music students (Yang et al., 2022).

#### 2.2.1 Perfectionism

The Chinese version of the Frost Multidimensional Perfectionism Scale (Frost et al., 1990) was used. Given the more stable association between maladaptive perfectionism and procrastination and anxiety (Sirois et al., 2017), this study selected two dimensions: “Focus on Errors” (9 items) and “Doubts about Actions” (4 items), totaling 13 items. A 5-point Likert scale (1 = completely disagree, 5 = completely agree) was used, with a total score range of 13–65 points. Higher scores indicated higher levels of maladaptive perfectionism. In this study, the Cronbach’s *α* coefficient of this scale was 0.87.

#### 2.2.2 Music performance anxiety

The Chinese version of the Kenny Music Performance Anxiety Scale (K-MPAI) developed by Kenny (2009) was used. This scale contains 40 items and measures the cognitive, affective, and physiological components of MPA. A 7-point Likert scale (0 = completely incorrect, 6 = completely correct) was used, with a total score range of 0–240. Higher scores indicated higher levels of anxiety about musical performance. In this study, the Cronbach’s α coefficient for this scale was 0.91.

#### 2.2.3 Self-efficacy

The Chinese version of the General Self-Efficacy Scale (GSES; Schwarzer and Jerusalem, 1995) was used. This scale was chosen based on the following considerations: (1) there is no validated Chinese version of existing music self-efficacy scales such as Ritchie and Williamon (2011); (2) General self-efficacy reflects stable beliefs across situations and is more suitable as a moderating variable at the personality level (Luszczynska et al., 2005). This scale contains 10 items, using a 4-point Likert scale (1 = completely incorrect, 4 = completely correct), with a total score range of 10–40. Higher scores indicated stronger self-efficacy. In this study, Cronbach’s *α* = 0.85.

#### 2.2.4 Practice procrastination

Based on Lay (1986) General Procrastination Scale and Tuckman (1991) Academic Procrastination Scale, a practice procrastination scale was developed by adapting the scale to the context of piano practice. This scale contains 8 items (see Supplementary A for the complete list), with example items such as “Even knowing I need to practice, I often make excuses to postpone it” and “I often start practicing a piece I’m about to play at the last minute.” A 5-point Likert scale (1 = completely disagree, 5 = completely agree) was used, with a total score ranging from 8 to 40. Higher scores indicate more severe procrastination. Exploratory factor analysis showed a single-factor structure, with a cumulative variance explained of 52.36%, and factor loadings for each item ranging from 0.58 to 0.78. The scale demonstrated good reliability (Cronbach’s α = 0.83, combined reliability CR = 0.88), with a mean variance extractable (AVE) of 0.49, close to the ideal threshold of 0.50. Combined with a CR significantly higher than the standard of 0.70, this indicates acceptable convergent validity (Fornell and Larcker, 1981). CFA was not conducted because the scale was developed and tested on the same sample, and performing CFA on identical data used for EFA does not constitute independent structural validation (Worthington and Whittaker, 2006). Splitting the sample was also considered but deemed suboptimal, as the resulting subsamples (n ≈ 78) would provide limited conditions for reliable parameter estimation given the moderate range of factor loadings observed. The composite reliability (CR = 0.88) and average variance extracted (AVE = 0.49) reported above provide supplementary evidence of convergent validity for the single-factor structure. Validation of this scale using an independent sample with standard CFA fit indices (e.g., CFI, TLI, RMSEA, SRMR) is recommended as a priority for future research. The correlation patterns between the scale and performance anxiety (*r* = 0.48) and self-efficacy (*r* = −0.39) were consistent with theoretical expectations, supporting criterion-related validity.

#### 2.2.5 Control variables

This study included gender, grade level, and years of piano study as control variables. These variables were chosen based on the following considerations: previous research has found that female music students generally have higher levels of performance anxiety than male students (Sokoli et al., 2022); students at different grade levels face different performance pressures and academic demands; years of piano study reflect students’ skill level and accumulated practice experience, which may influence their practice behavior patterns. Controlling for these variables helps to more accurately estimate the relationships between the core variables.

### 2.3 Research procedure

This study was approved by the Ethics Committee of Jinzhong University (Approval No.: JZXY-2025-EC-268). Data collection took place from March to April 2025. The study was conducted via online questionnaire, with the questionnaire link distributed through WeChat and QQ groups for students in university music departments. An informed consent form was included at the beginning of the questionnaire, explaining the research purpose, the principle of voluntary participation, and the guarantee of anonymity. Participants could only access the formal questionnaire after clicking “agree.” To control for common method bias, the questionnaire adopted anonymous responses and emphasized that there were no right or wrong answers. Each scale had independent instructions, and an attention test question was included in the middle to identify invalid responses. The questionnaire took approximately 10–15 min to complete, and participants received a 5 RMB WeChat red envelope reward upon completion.

### 2.4 Data analysis

Data analysis was performed using SPSS 26.0. First, Harman’s one-way test for common method bias was used. Second, descriptive statistics and Pearson correlation analysis were conducted. Then, the moderated mediation effect was tested using the PROCESS macro program (Model 14; Hayes and Rockwood, 2020; Hayes, 2017; Preacher et al., 2007), the specific syntax of which is given in Supplementary B. All continuous variables were z-standardized prior to analysis so that all regression coefficients would be on a common standardized metric, facilitating comparison of relative effect sizes across variables with different original scales. Parameter estimation was performed using the bootstrap method, with 5,000 repeated samplings. The significance of the effect was determined by whether the 95% bias-corrected confidence interval contained 0. Finally, a simple slope analysis was conducted to examine the differences in the mediation effect at different levels of self-efficacy.

## 3 Results

### 3.1 Common method bias test

This study used a combination of procedural controls and statistical tests to control for common method bias. For procedural controls, anonymous responses were used to reduce social desirability bias, independent instructions were provided for each scale to create psychological isolation, and an attention test question was included in the middle of the questionnaire to identify invalid responses. For statistical tests, Harman’s one-factor test was used for diagnosis. All measurement items were included in exploratory factor analysis. The results showed that the variance explained by the first factor without rotation was 28.63%, lower than the critical value of 40% (Podsakoff et al., 2012), indicating that there was no serious common method bias problem in this study.

### 3.2 Descriptive statistics and correlation analysis

The means, standard deviations, and correlation coefficients of each variable are shown in Table 1. Descriptive statistics show that the participants’ perfectionism score was 42.35 (*SD* = 8.72), which is above average on the scale; their performance anxiety score was 78.42 (*SD* = 22.56), which is below average, indicating a certain degree of performance anxiety in the sample; their self-efficacy score was 27.83 (*SD* = 5.14), which is moderate; and their practice procrastination score was 24.67 (*SD* = 6.38), indicating that practice procrastination is relatively common in the sample.

**Table 1:** Descriptive statistics and correlation matrix of study variables (*N* = 156).

Variable	*M*	*SD*	1	2	3	4
1. Perfectionism	42.35	8.72	1			
2. Music performance anxiety	78.42	22.56	0.52**	1		
3. Self-efficacy	27.83	5.14	−0.23**	−0.45**	1	
4. Practice procrastination	24.67	6.38	0.41**	0.48**	−0.39**	1

**p < 0.01.

Correlation analysis showed that perfectionism was moderately positively correlated with performance anxiety (*r* = 0.52, *p* < 0.01) and with practice procrastination (*r* = 0.41, *p* < 0.01), and weakly negatively correlated with self-efficacy (*r* = −0.23, *p* < 0.01). Music performance anxiety was moderately positively correlated with practice procrastination (*r* = 0.48, *p* < 0.01) and moderately negatively correlated with self-efficacy (*r* = −0.45, *p* < 0.01). Self-efficacy was moderately negatively correlated with practice procrastination (*r* = −0.39, *p* < 0.01). These results are consistent with the research hypotheses, and the correlation coefficients among all variables are below 0.70, indicating the absence of severe multicollinearity and providing preliminary support for further testing of the hypothesized associations.

### 3.3 Moderated mediation effect test

**Figure 1:**
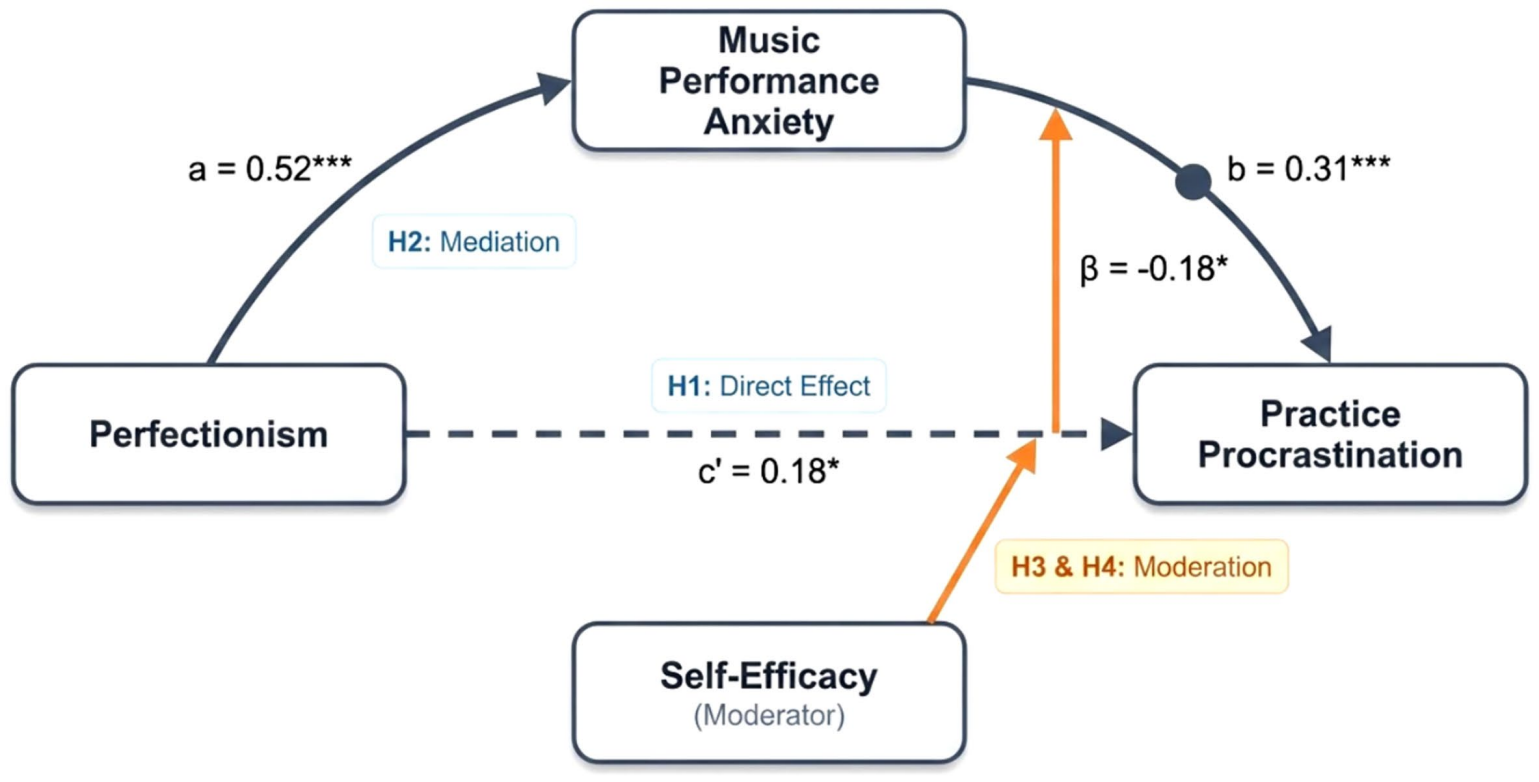
Results of the moderated mediation model. Standardized coefficients shown. Dashed line indicates direct effect. *p < 0.05, **p < 0.01, ***p < 0.001.

Controlling for gender, grade level, and years of piano study, the moderated mediation effect was tested using the PROCESS macro program Model 14. The results are shown in Table 2 and Figure 1. In the first regression step, perfectionism significantly and positively predicted performance anxiety (*α* = 0.52, *t* = 7.52, *p* < 0.001). The model explained 28% of the variance of performance anxiety (*R^2^* = 0.28), indicating that perfectionism is an important predictor of performance anxiety, and the first stage (α path) of the mediation effect was validated. Among the control variables, gender, grade level, and years of piano study did not have significant predictive effects on performance anxiety (*ps* > 0.05).

**Table 2:** Results of the moderated mediation model.

Regression equation	Outcome variable	Predictor	β	SE	*t*	*p*
Step 1	Music performance anxiety	Constant	0.00	0.07	0.00	1.000
		Perfectionism	0.52	0.07	7.52	< 0.001
		Gender	−0.08	0.07	−1.14	0.256
		Grade	0.05	0.07	0.71	0.479
		Years of piano training	−0.03	0.07	−0.43	0.668
		*R^2^* = 0.28, *F*(4, 151) = 14.68, *p* < 0.001				
Step 2	Practice procrastination	Constant	0.00	0.06	0.00	1.000
		Perfectionism	0.18	0.07	2.57	0.011
		Music performance anxiety	0.31	0.08	3.87	< 0.001
		Self-efficacy	−0.21	0.07	−3.00	0.003
		Anxiety × Self-efficacy	−0.18	0.07	−2.57	0.011
		Gender	−0.05	0.06	−0.83	0.408
		Grade	0.02	0.06	0.33	0.742
		Years of piano training	0.04	0.06	0.67	0.504
		*R^2^* = 0.42, *F*(7, 148) = 15.32, *p* < 0.001				

β = standardized regression coefficient. All predictor and outcome variables were z-standardized (M = 0, SD = 1) prior to analysis, which accounts for the intercepts of 0.00 in both regression equations.

In the second regression step, the direct effect of perfectionism on procrastination was significant (c’ = 0.18, *t* = 2.57, *p* = 0.011), indicating that even after controlling for performance anxiety, perfectionism can still independently predict procrastination, supporting Hypothesis 1. Music performance anxiety significantly and positively predicted practice procrastination (*β* = 0.31, *t* = 3.87, *p* < 0.001), while self-efficacy significantly and negatively predicted practice procrastination (*β* = −0.21, *t* = −3.00, *p* = 0.003). More importantly, the interaction term between music performance anxiety and self-efficacy significantly predicted practice procrastination (*β* = −0.18, *t* = −2.57, *p* = 0.011), suggesting that self-efficacy may moderate the relationship between music performance anxiety and practice procrastination. This model explained 42% of the variance in practice procrastination (*R^2^* = 0.42), demonstrating moderate to high explanatory power, and Hypothesis 3 is preliminarily supported.

### 3.4 Simple slope analysis

**Figure 2:**
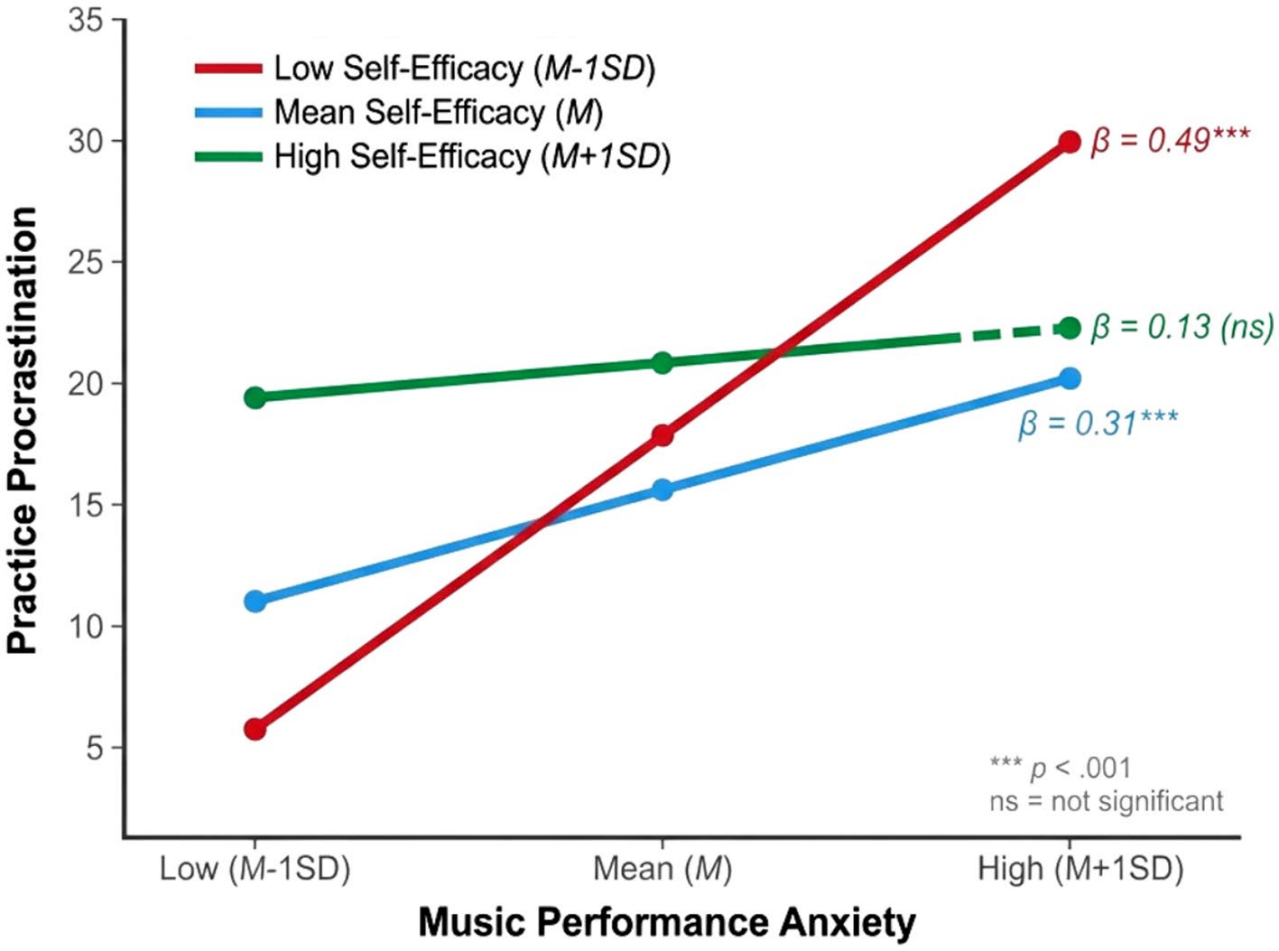
Simple slopes of music performance anxiety predicting practice procrastination at different levels of self-efficacy. Lines represent regression slopes at low (*M*-1*SD*), mean (*M*), and high (*M* + 1*SD*) levels of self-efficacy.

To further explain the specific pattern of the moderating effect, a simple slope analysis was conducted to examine the predictive effect of music performance anxiety on practice procrastination at different levels of self-efficacy (*M*-1*SD*, *M*, *M* + 1*SD*). The results are shown in Table 3 and Figure 2. At low self-efficacy levels, music performance anxiety had a strong and significant positive predictive effect on practice procrastination (*β* = 0.49, *t* = 4.90, *p* < 0.001); at moderate self-efficacy levels, this predictive effect weakened but remained significant (*β* = 0.31, *t* = 3.87, *p* < 0.001); at high self-efficacy levels, this predictive effect was the weakest and no longer significant (*β* = 0.13, *t* = 1.44, *p* = 0.152). From the perspective of effect size changes, the slope of the low self-efficacy group (0.49) was approximately 3.8 times that of the high self-efficacy group (0.13), indicating that self-efficacy has a practical significance in moderating the anxiety-procrastination relationship. As shown in Figure 2, the three regression lines exhibit a clear fan-shaped distribution, with the steepest slope under the low self-efficacy condition, while the slope under the high self-efficacy condition tends to be gentler. These results indicate that high self-efficacy effectively weakens or even eliminates the positive effect of music performance anxiety on practice procrastination, further supporting Hypothesis 3.

**Table 3:** Simple slopes of music performance anxiety predicting practice procrastination at different levels of self-efficacy.

Self-efficacy level	β	SE	*t*	*p*
Low (*M* − 1*SD*)	0.49	0.10	4.90	< 0.001
Mean (*M*)	0.31	0.08	3.87	< 0.001
High (*M* + 1*SD*)	0.13	0.09	1.44	0.152

β = standardized regression coefficient.

### 3.5 Conditional indirect effect test

To test the moderated mediation effect, the conditional indirect effect of perfectionism influencing practice procrastination through music performance anxiety was further examined at different levels of self-efficacy. The results are shown in Table 4. At low self-efficacy levels, the indirect effect was 0.25 (95% CI = [0.15, 0.38]), with a confidence interval not including 0, indicating a significant and large mediation effect. At moderate self-efficacy levels, the indirect effect was 0.16 (95% CI = [0.09, 0.25]), also indicating a significant mediation effect. At high self-efficacy levels, the indirect effect was 0.07 (95% CI = [−0.02, 0.17]), with a confidence interval including 0, indicating that the mediation effect was no longer significant. These results indicate that as self-efficacy levels increase, the indirect effect of perfectionism influencing practice procrastination through music performance anxiety gradually weakens and eventually disappears, exhibiting a clear gradient change pattern.

**Table 4:** Conditional indirect effects at different levels of self-efficacy.

Self-efficacy level	Indirect effect	Boot SE	95% CI LL	95% CI UL
Low (*M* − 1*SD*)	0.25	0.06	0.15	0.38
Mean (*M*)	0.16	0.04	0.09	0.25
High (*M* + 1*SD*)	0.07	0.05	−0.02	0.17
Index of moderated mediation	−0.09	0.04	−0.17	−0.03

Indirect effect = a × b, where a represents the effect of perfectionism on music performance anxiety (0.52), and b represents the conditional effect of music performance anxiety on practice procrastination at each level. Bootstrap resampling = 5,000. CI, confidence interval; LL, lower limit; UL, upper limit.

The moderated mediation index was −0.09 (Boot SE = 0.04, 95% CI = [−0.17, −0.03]), and the 95% confidence interval did not include 0, indicating a significant moderated mediation effect. This means that the indirect effect of perfectionism on practice procrastination through music performance anxiety is moderated by self-efficacy, thus supporting hypotheses 2 and 4.

In summary, the observed patterns in this cross-sectional study are consistent with all four hypothesized associations, though the design does not permit causal inference: perfectionism significantly and positively predicts practice procrastination (H1); MPA may serve as an intermediary variable in the association between perfectionism and practice procrastination (H2); self-efficacy moderates the relationship between music performance anxiety and practice procrastination (H3); and self-efficacy moderates the second segment of the indirect pathway from perfectionism to practice procrastination through MPA, as supported by the moderated mediation analysis (H4).

## 4 Discussion

### 4.1 The relationship between perfectionism and practice procrastination

The results indicated that perfectionism was positively associated with practice procrastination, consistent with H1. This is consistent with the findings of Flett et al. (1992), Steel (2007), and Sirois et al. (2017) in the field of general procrastination. As mentioned in the introduction, the core of maladaptive perfectionism is an excessive focus on errors and doubt about action (Frost et al., 1990; Hewitt and Flett, 1991). When the expected practice results cannot meet high standards, students may choose to postpone practice to avoid facing imperfect results. As Yosopov et al. (2024) pointed out, fear of failure and failure generalization are important mechanisms by which perfectionism leads to procrastination. This study extends the relationship between perfectionism and procrastination to the field of music practice, echoing Stoeber and Eismann (2007) distinction between adaptive and maladaptive perfectionism, and consistent with the findings of Yang et al. (2022) among Chinese piano students. Combined with the rising trend of perfectionism among the younger generation (Curran and Hill, 2019), the results of this study have theoretical value in understanding the behavioral consequences of perfectionism. Notably, piano practice is characterized by immediate feedback—students can immediately hear their mistakes during practice. This immediacy may make perfectionists more intolerant of imperfections during practice, thus exacerbating their procrastination tendencies. This situational characteristic makes the association between perfectionism and practice procrastination potentially more pronounced in piano learning than in other academic fields.

### 4.2 The role of music performance anxiety as an intermediary variable

The results indicated that MPA may function as an intermediary variable in the association between perfectionism and practice procrastination, with the conditional indirect effect being significant at the mean level of self-efficacy, consistent with H2. This addresses the core question in the introduction: “How does perfectionism influence practice procrastination?” This result can be understood through the cognitive-emotion-behavioral theoretical chain: maladaptive perfectionists’ excessive focus on errors and stringent standards for performance (cognitive level) make them more likely to perceive the potential threat of failure when faced with practice tasks, thus triggering music performance anxiety (emotional level); while individuals in an anxious state tend to adopt avoidance strategies to obtain short-term emotional relief (Sirois and Pychyl, 2013), and practice procrastination is a specific manifestation of this avoidance behavior (behavioral level). The avoidance behavior examined in this study occurs specifically during the preparation phase, when students postpone the deliberate practice needed to develop performance readiness, rather than during actual performance or post-performance evaluation. Previous studies have consistently confirmed that perfectionism predicts MPA (Kenny, 2011; Kenny et al., 2004; Kobori et al., 2011; Patston and Osborne, 2016; Dobos et al., 2019; Gök and Yalçınkaya-Alkar, 2025).

Regarding the relationship between anxiety and procrastination, Biasutti and Concina (2014) found that coping strategies significantly predicted MPA levels; Passarotto et al. (2023) found that piano students with higher anxiety levels required more practice time to achieve satisfactory performances, which may reflect the negative impact of anxiety-induced avoidance and procrastination on practice efficiency; Sokoli et al. (2022) confirmed that pre-competition anxiety predicts self-rated performance quality; and Irie et al. (2023) described in detail the symptom manifestations of MPA at different stages before, during, and after performances, indicating that anxiety may begin to affect students’ behavioral choices during the practice phase. This study empirically tested this mechanism, suggesting that MPA may function as an emotional intermediary through which perfectionism is associated with practice procrastination. This finding suggests that interventions for practice procrastination should not only focus on the procrastination behavior itself, but also identify and address its underlying emotional root cause: performance anxiety.

### 4.3 Moderating role of self-efficacy

This study found that self-efficacy significantly modulates the relationship between MPA and practice procrastination, supported by H3 and H4. Simple slope analysis revealed that the positive association between MPA and practice procrastination was strongest at low self-efficacy levels, weaker at moderate levels, and no longer statistically significant at high self-efficacy levels. This addresses the question posed in the introduction: “Which factors modulate this process?”.

These results are consistent with Bandura (1977) self-efficacy theory. Seo (2008) and Huang et al. (2023) confirmed the protective role of general self-efficacy from a procrastination perspective; González et al. (2018) and Wang and Yang (2024) also verified the buffering effect of self-efficacy on the negative effects of anxiety in the field of music. Notably, this study, using a generalized self-efficacy scale, discovered a significant moderating effect, indicating that even generalized beliefs in ability can provide psychological protection for music students. This expands upon previous research focusing on domain-specific music self-efficacy (McPherson and McCormick, 2006; Ritchie and Williamon, 2011).

As discussed in the introduction, self-efficacy is theorized to shape the behavioral coping choices individuals make when experiencing anxiety, influencing whether they engage in approach-oriented strategies or resort to avoidance. Students with high self-efficacy, even when feeling anxious, still believe in their ability to overcome difficulties and are therefore more inclined to adopt problem-focused strategies rather than avoidance and procrastination. Arbinaga Ibarzábal (2023) found that resilient behavior in music students is related to self-efficacy, and a systematic review by Varela et al. (2016) also emphasized the central role of self-regulation in music learning. Although self-efficacy was significantly correlated with MPA at the bivariate level (*r* = −0.45), the theoretical model positioned self-efficacy as moderating the behavioral response to anxiety rather than the generation of anxiety itself. Future research could examine whether self-efficacy also attenuates the perfectionism–MPA link in alternative model specifications. From a practical perspective, this finding has important implications: even if it is impossible to quickly change students’ perfectionist tendencies or completely eliminate their anxiety experiences, enhancing self-efficacy can still serve as an effective intervention entry point to help students break the vicious cycle of anxiety and procrastination.

### 4.4 Research contributions

This study makes the following theoretical and practical contributions. At the theoretical level, this study introduces “practice procrastination” as an independent construct into music education research, filling a gap in the field’s focus on dysfunctional practice behaviors and responding to McPherson (2022) call for a comprehensive perspective in music performance research. The findings are also consistent with a psychological mechanism in which perfectionism is associated with practice procrastination, with MPA appearing to function as an intermediary variable in this relationship, integrating Kenny (2011) MPA theoretical framework and Sirois and Pychyl (2013) procrastination emotion regulation theory from a cognitive-emotion-behavioral chain. This integration not only addresses the lack of research on the behavioral consequences of MPA in music psychology but also provides a new application context for procrastination research, promoting dialog and integration between the two research fields. The study further clarifies the boundary conditions of self-efficacy as a potential protective factor, indicating that general self-efficacy can also buffer the negative effects of anxiety, expanding the research perspective that previously focused on domain-specific self-efficacy. On a practical level, this study provides music educators with screening criteria for identifying high-risk students (high perfectionism, high anxiety, and low self-efficacy) and points the way for developing targeted intervention strategies. Research in educational performing arts contexts has similarly demonstrated the value of identifying distinct student profiles based on performance anxiety and emotional characteristics, enabling targeted pedagogical interventions (Mastrothanasis et al., 2023). Specifically, teachers can use a short questionnaire to assess students’ characteristics across these three dimensions and provide early intervention for students in the high-risk range, thereby preventing the problem of procrastination in practice from worsening.

### 4.5 Limitations and future directions

This study has several limitations that warrant consideration. The cross-sectional design precludes causal inference, and the use of convenience sampling limits the generalizability of the findings. The sample size justification was based on guidelines developed for simple mediation models (Fritz and MacKinnon, 2007), and no dedicated power analysis for the moderated mediation model (PROCESS Model 14) was performed, which represents a methodological limitation. All data were collected via self-report from a single source, and although procedural controls and Harman’s one-factor test did not indicate serious common method bias, shared method variance cannot be entirely excluded. The Practice Procrastination Scale was developed and evaluated using EFA within the same sample, and CFA with an independent sample was not performed, which limits the evidence for structural validity. The sample was drawn exclusively from Chinese undergraduate piano students, and cultural factors related to educational values, parental expectations, and attitudes toward mental health may limit the applicability of these findings to other cultural contexts. The General Self-Efficacy Scale was used rather than a domain-specific music self-efficacy measure, which may not capture efficacy beliefs directly tied to piano practice. Other potential moderating variables, such as social support (Huawei and Salarzadeh Jenatabadi, 2024), were not examined in this study.

Several directions for future research emerge from these limitations. Longitudinal and experimental designs with larger and more diverse samples would allow testing of causal directionality, as persistent anxiety experiences may reinforce perfectionistic cognitions over time, and procrastination itself may exacerbate anxiety in a reciprocal manner. Alternative moderated structures, such as self-efficacy moderating the perfectionism-to-anxiety pathway rather than the anxiety-to-procrastination pathway, also warrant investigation. Future studies could incorporate objective practice data such as digital practice logs (Suzuki et al., 2024) and adopt validated music self-efficacy instruments (Ritchie and Williamon, 2011) to address measurement limitations. The Practice Procrastination Scale should be validated using an independent sample with standard CFA fit indices. The cognitive-emotional-behavioral framework examined in this study may also be applicable to other performing arts domains such as theater and dance, as well as visual arts contexts where public exhibition creates comparable evaluative pressure, and extending this model across artistic disciplines would strengthen its contribution to the broader field of Performance Science. Intervention studies are also needed to verify whether enhancing self-efficacy can effectively reduce the anxiety-procrastination association in practice settings.

## 5 Conclusion

This cross-sectional study used a moderated mediation model to examine the associations among perfectionism, MPA, self-efficacy, and practice procrastination in 156 piano major students from Chinese universities. The findings are consistent with the following patterns: (1) perfectionism is positively associated with practice procrastination; (2) MPA appears to function as an intermediary variable in this association; (3) self-efficacy moderates the link between MPA and procrastination, with higher self-efficacy attenuating or eliminating the positive association between anxiety and procrastination; and (4) the indirect pathway from perfectionism to procrastination through MPA is conditional on self-efficacy level, as indicated by the moderated mediation analysis. The research findings reveal the formation mechanism and protective factors of procrastination in piano students, providing empirical evidence for music educators to identify high-risk students and develop targeted intervention strategies. Specifically, music educators should pay attention to students’ perfectionist tendencies, guiding them to shift their focus from “avoiding mistakes” to “continuous progress,” and helping them develop a growth-oriented practice mindset. To manage performance anxiety, evidence-informed approaches such as graded exposure to performance settings, cognitive restructuring, and relaxation techniques have shown promise in the MPA intervention literature (Burin and Osório, 2016), though their efficacy in reducing practice procrastination specifically remains to be tested. Simultaneously, setting achievable phased goals, providing timely positive feedback, and encouraging students to record their progress can enhance their self-efficacy. Students exhibiting high perfectionism, high anxiety, and low self-efficacy should receive particular attention and support.

## Data Availability

The original contributions presented in the study are included in the article/Supplementary material, further inquiries can be directed to the corresponding author.
