# Supplementary material for: Perfectionism and practice procrastination in piano majors: a moderated mediation model of music performance anxiety and self-efficacy
Source: Front Psychol. 2026 May 14;17:1786554. doi: 10.3389/fpsyg.2026.1786554 (PMC13216481; doi:10.3389/fpsyg.2026.1786554)
Supplement: Supplementary file 1 [file Supplementary_File_1.docx]

Supplementary Material

**Supplementary A.** Practice Procrastination Scale

Please rate the following statements according to your actual situation:

1 = Completely disagree, 2 = Somewhat disagree, 3 = Unsure, 4 = Somewhat agree, 5 = Completely agree

1. Even knowing I need to practice, I often find excuses to postpone it.

2. I often start practicing pieces I'm about to play at the last minute.

3. I spend my practice time on other, less important things.

4. I often regret procrastinating on practice.

5. I find it difficult to start practicing according to my schedule.

6. When I think about what I need to practice, I feel reluctant to start.

7. Even when I have enough time, I postpone practicing difficult sections.

8. I often tell myself, "I'll practice tomorrow."

**Supplementary B.** Data Analysis Syntax

SPSS PROCESS Macro Syntax (Model 14):

PROCESS

y=Practice Procrastination

/x=Perfectionism

/m=Musical Performance Anxiety

/w=Self-Efficacy

/model=14

/boot=5000

/seed=12345

/covcoeff=1

/jn=1

/cov=Gender Grade Years of Piano Learning
